# Transurethral resection of prostate syndrome: report of a case

**DOI:** 10.11604/pamj.2013.14.14.1906

**Published:** 2013-01-09

**Authors:** Brahim Boukatta, Hicham Sbai, Ferdaous Messaoudi, Zakaria Lafrayiji, Abderrahim El Bouazzaoui, Nabil Kanjaa

**Affiliations:** 1 Department of Anesthesia and critical care Medicine, Hassan II University Hospital, Fez, Morocco

**Keywords:** Transurethral resection, TURP syndrome, hyponatremia, hypertonic saline, coma

## Abstract

We report a case of transurethral resection of prostate (TURP) syndrome. A 78-year-old man with prostatic hypertrophy was scheduled for transurethral resection of the prostate under spinal anesthesia. 30 minutes after the end of the surgery, the patient presented signs of TURP syndrome with bradycardia, arterial hypotension, cyanosis, hypoxemia and coma. The electrolytes analysis revealed an acute hyponatremia (sodium concentration 125 mmol/L). Medical treatment consisted of hypertonic saline solution 3%, volume expansion, intubation and ventilation. The presented case describes a typical TURP syndrome, which was diagnosed and treated early. The patient was discharged from hospital without any complications.

## Introduction

Transurethral resection of prostate (TURP) syndrome is a systemic complication of transurethral resection of the prostate or bladder tumours, caused by excessive absorption of electrolyte-free irrigation fluids [1]. This syndrome may potentially cause neurologic disturbance, pulmonary edema, cardiovascular compromise, and death [2]. Normal saline cannot be used as irrigation solution with conventional monopolar resection [3]. Glycine solution is almost universally used as an irrigation solution in traditional therapeutic endoscopic urologic procedures [4]. The incidence of this complication is between 0.78% and 1.4% [5]. The surgeon should be informed immediately, the intervention stopped as quick as possible and the treatment should start without delay. We report the case of a 78-year-old male patient, who presented a severe TURP syndrome 30 minutes after surgery with moderate hyponatremia.

## Patient and observation

A 78-year-old man, without medication coexisting diseases, body weight 50 Kg, underwent transurethral resection of prostate under spinal anesthesia, with 2.5 ml of hyperbaric bupivacaine (0.5%). That extended to the T10 level, as tested by pin prick. The heart rate at the start of the operation was 85 bpm, blood pressure 140/80 mmHg, electrocardiogram normal and spo2 was 98%. The patient was positioned in lithotomy posture. And the TURP surgery was started. The preoperative values of serum sodium was 145 mmol/L, potassium 4.1 mmol/L, creatinine 14 mg/L and urea 0.4g/L. A routine monitoring of fluid absorption by expired breath tests is not practiced in our hospital. The irrigation fluid used by urologist was glycine 1.5%. TURP was performed by monopolar instrument. The height of the irrigating fluid reservoir was fixed at 60 cm height from patient's bed. The total intraoperative bleeding was estimed at 500 ml. The average peroperative blood pressure was 145/85 mmHg and heart rate 85 bpm. During surgery, which lasted for 120 min, the patient had been given 1500 ml normal saline solution by intravenous infusion. 30 min after the end of operation, patient suddenly was developed in recovery room, nausea, vomiting, agitation then rapidly coma, hypoxemia (spO2 70%), hypotension (70/40 mmHg) and bradycardia (45 bpm). The abdomen and lung auscultation were normal. He was intubated and ventilated with an FIO2 of 1. He was loaded with 500 ml of hydroxyethyl starch and 500 ml of saline 0.9%, a central venous line was inserted, the inotopic support (norepinephrine) was administred, inducing normalization hemodynamic. The TURP syndrome was subsequently confirmed by an immediately electrolyte analysis revealed a decrease in serum sodium concentration from 145 mmHg to 125 mmHg. Hypertonic saline solution 3% at a rate of 50 ml/h was started. The patient was transfused with 2 units blood. Furosemide wasn't given. The measurement of all irrigating fluids showed that four litres had been absorbed. Blood ammonemia and glycinemia concentrations weren't measured. The patient was transferred sedated and ventilated to the intensive care unit. The blood pressure and heart rate were gradually stabilized at 120/70 and 80 bpm. A chest x-ray of the lungs and ECG were normal. Three hours later, the sodium concentration was 129 mmHg, potassium 3.2 mmHg, glucose level 2.31g/l, cardiac Troponin 0.2ng/ml, creatinine 10mg/l and hemoglobin 9.7g/dl. The second postoperative day, sodium concentration was increased to 134 mmol/L, potassium 4.7 mmol/L, hemoglobin 12.7g/l and Troponin 3.132 ng/ml. The patient was extubated 2 days later. The concentration sodium level stabilized at 135 mmol/L, potassium 3.5 mmol/L and Troponin 0.15 ng/ml. He was returned to the urologic clinic six days later and he was discharged from the hospital 10 days later in good condition.

## Discussion

Transurethral resection syndrome during transurethral resection of the prostate (TURP) results from excessive absorption of electrolyte-free irrigation fluids causing acute hypervolemia and hyponatremia [6, 7]. The clinical spectrum ranges from asymptomatic hyponatremia to electrocardiographic changes, nausea, vomiting, convulsions, coma, alterations of vision, pulmonary edema, cardiovascular compromise and death [8]. The role of irrigation solution is to distend the bladder, clear the surgical site and wash away resected tissue and blood. Various irrigation fluids (Glycine, sorbitol, mannitol and normal saline) have been used for TURP. Glycine solution is the most commonly used irrigant in traditional therapeutic endoscopic urologic procedures [9]. Several reports showed that Glycine absorption causes echocardiogram changes, it is associated with subacute effects on the myocardium, as T-wave depression or inversion on electrocardiography for up to 24 hours after surgery, increased Troponin [10]. Our patient presented 30 minutes after the end of intervention a typical severe TURP syndrome in spite of a moderate decrease of sodium concentration. The severity of our patient's clinical picture, in contrast with moderately hyponatremia, could be explained by multiples factors: age, rapidly decrease of sodium concentration, hyperammonemia and probably hyperglycinemia. The hypotension was to be secondary to bleeding. Effectively, in TURP syndrome, the hypertension may be absent if bleeding is important [11, 12], and on the pathophysiology, the hyponatremia may lead to net water flux along osmotic out of the intravascular space inducing hypovolemic shock [13]. The release of endotoxins into the circulation and the associated metabolic acidosis may also contribute to the hypotension. The diagnosis of TURP syndrome should be rapid, so, the spinal anesthesia is considered to be the anesthetic technique of choice, allowing early detection of neurological symptoms (patient awake). The treatment of severe TURP syndrome with hypoxemia and shock is based on supporting respiration (intubation, ventilation), adrenergic drugs, plasma volume expansion, hypertonic saline 3%, between 50 and 100 ml/h. The diuretic therapy is not recommended in a hemodynamically unstable patient and surgery should be terminated as quick as possible. Several new procedures, such as laser ablation, laser enucleation, photoselective vaporization and bipolar resection in saline may be a good surgical alternative for preventing this complication especially in critically ill patients [14].

## Conclusion

The systemic absorption of such an irrigating fluid may be associated with serious complications. The best prevention method can be obtained by adopting a correct surgical technique and optimizing the patient's conditions preoperatively. At the moment, the bipolar resection in saline and laser TURP may be a good alternative procedure.

## References

[1] Robert G, Hahn MD (1995). Transurethral resection syndrome after transurethral resection of bladder tumours. Can J Anaesth..

[2] Reich O, Gratzke C, Bachmann A, Seitz M (2008). Morbdity, mortality and early outcome of transurethral resection of the prostate: Aprospective multicenter evaluation of 10654 patients. J Urol..

[3] Imiak S, Weavind L, Dabney T, Wenker O (1999). TURP Syndrome - interactive Case Report in Anesthesia and Critical Care. The Internet Journal of Anesthesiology.

[4] Hawary A, Mukhtar K, Sinclair A, Pearce I (2009). Transurethral resection of the prostate syndrome: almost gone but not forgotten. J Endourol..

[5] Zepnick H, Steinbach F, Schuster F (2008). Value of transurethral resection of the prostate (TURP) for treatment of symptomatic benign prostatic obstruction (BPO): an analysis of efficiency and complications in 1015 cases. Aktuelle Urol..

[6] Rassweiler J, Teber D, Kuntz R, Hofmann R (2006). Complication of transurethral resection of the prostate (TURP)-incidence, management, and prevention. Eur Urol..

[7] Tolksdorf W, Goetz D, Peters HJ, Potempa J, Lutz H (1980). Infusion therapy during transurethral prostatectomy. Infusionsther Klin Ernahr..

[8] Gupta K, Rastogi B, Jain M, Gupta PK, Sharma D (2010). Electrolyte changes: an indirect method to assess irrigation fluid absorption complication during transurethral resection of prostate: A prospective study. Saudi J Anaesth..

[9] Yousef A, Suliman G, Elashry O, Elshorahy M, Elgamasy A (2010). A randomized comparison between three types of irrigating fluids during transurethral resection in benign prostatic hyperplasia. BMC Anesthesiology.

[10] Collins JW, Macdermott S, Brad RA, Keely FX, Timoney AG (2007). The effects of the choice of irrigating fluid on cardiac stress during Transurethral resection of the prostate: A comparison between 1.5% Glycine and 5% glucose. J Urol..

[11] Harrison RH, Boren JS, Robinson JR (1956). Dilutional hyponatremic shock: Another concept of the transurethral prostatic resection reaction. J Urol..

[12] Hahn RG (1989). Acid phosphatase levels in serum during transurethral prostatectomy. Br J Urol..

[13] Ceccarelli FE, Mantell LK (1961). Studies on fluid and electrolyte alterations during transurethral prostatectomy. J Urol..

[14] Wang JM, Creel DJ, Wong KC (1989). Transurethral resection of the prostate, serum Glycine levels, and ocular evoked potentially. Anesthesiology..

